# Draft Genome Sequence of the Bacterium Delftia acidovorans Strain D4B, Isolated from Soil

**DOI:** 10.1128/MRA.00635-21

**Published:** 2021-11-04

**Authors:** Jackson Harris, Marie Gross, Jeremy Kemball, Sanaz Farajollahi, Patrick Dennis, John Sitko, J. Jordan Steel, Erin Almand, Nancy Kelley-Loughnane, Vanessa A. Varaljay

**Affiliations:** a Department of Biology, United States Air Force Academy, Colorado Springs, Colorado, USA; b UES, Inc., Dayton, Ohio, USA; c University of Dayton, Dayton, Ohio, USA; d Soft Matter Materials Branch, Materials and Manufacturing Directorate, Air Force Research Laboratory, Wright-Patterson Air Force Base, Ohio, USA; University of Maryland School of Medicine

## Abstract

Delftia acidovorans strain D4B is an aerobic bacterium within the *Betaproteobacteria* lineage that was isolated from soil. The genome size is 6.26 Mbp, with a G+C content of 67%. The genome encodes enzymes potentially involved in the degradation of fluorinated compounds.

## ANNOUNCEMENT

Here, we describe the draft genome sequence of Delftia acidovorans strain D4B, which was isolated from soil contaminated with perfluorinated compounds (PFCs). Delftia acidovorans strains are environmental bacteria with diverse metabolic pathways that make them strong candidates for bioremediation (1). D. acidovorans strain D4B grows aerobically in the presence of either 1 ppm or 100 ppm of the perfluoroalkyl substance (PFAS) perfluorooctanoic acid (PFOA) in Raymond’s medium (2) supplemented with 1,000 ppm glucose at 30°C with shaking. The sequenced genome of D. acidovorans strain D4B provides the first genome for an isolate of this genus, which will further our understanding of how this organism could be leveraged for bioremediation.

D4B was isolated from a Winogradsky column with soil from a PFC-contaminated site. The soil was incubated with paper, hard-boiled egg yolk, and lake water artificially contaminated with either perfluorooctane sulfonate (PFOS) (100 ppm or 666 ppm) or PFOA (100 ppm or 750 ppm). Columns were grown for 1 month, and the top of the culture was swabbed. Unique colonies were restreaked on 100 ppm PFOA-agar plates, and D4B was isolated as a single colony. D4B was grown in tryptic soy broth at 32°C for 18 h with shaking. The culture was diluted, grown for ∼8 h under identical conditions, and harvested in the mid-logarithmic growth phase. Genomic DNA was extracted from the harvested cell pellet using the Qiagen DNeasy UltraClean microbial silica spin column kit. The library was made with 155 ng of DNA using Illumina DNA Prep, (M) Tagmentation (24 sample, product number 20018704; Illumina) and IDT for Illumina DNA UD indexes. The library was sequenced using the NovaSeq 6000 SP reagent kit v1.5 (200 cycles) (product number 20040719; Illumina) on an Illumina NovaSeq 6000 system run using 101-bp paired-end (PE) reads.

Default parameters were used for all software unless otherwise specified. Sequencing resulted in 4,482,539 raw PE reads, which were trimmed and quality-filtered with Trimmomatic v0.39 (3). The 4,472,394 (894,478 total) trimmed, quality-filtered PE and unpaired reads (average read length, 101 bp) were assembled using SPAdes 3.13.1 (4) in careful mode, resulting in ∼140× coverage and 52 contigs (≥200 bp), with a G+C content of 67%, *N*_50_ value of 334,654 bp, *L*_50_ value of 6, and genome size of 6,259,045 Mb, as calculated by QUAST v5.0.2 (5). D4B genome completeness was estimated to be 99.85%, with <1% contamination, using CheckM v1.1.3 (6). Whole-genome, *de novo* gene prediction and annotation were performed with Prokka v1.12 (7), which resulted in 5,484 putative genes of >50 amino acids. RNAmmer v1.2 (8) was used to extract the full-length 16S rRNA gene, which was 100% identical to other Delftia acidovorans sequences using BLASTN against the NCBI nonredundant database.

Based on the Prokka annotations, BLASTP searches with two Delftia acidovorans haloacid dehalogenases from the NCBI database (GenBank accession numbers WP_011137954.1 and PZP66635.1), and Phyre2 homology-based structural predictions (9) with 100% confidence scores, we identified two putative haloacid dehalogenases and a fluoroacetate dehalogenase, which could be involved in the degradation of fluorinated compounds. This sequenced genome should provide critical insights into the metabolism and physiology leading to degradation and remediation of fluorinated compounds by this ubiquitous soil bacterium.

### Data availability.

This whole-genome shotgun project has been deposited in DDBJ/ENA/GenBank under the accession number JAHLGQ000000000. The version described in this paper is version JAHLGQ000000000.2. The raw reads have been deposited in the Sequence Read Archive (SRA) database under SRA accession number SRP323477 and BioProject accession number PRJNA736486.
