# Erratum for Harris et al., “Draft Genome Sequence of the Bacterium Delftia acidovorans Strain D4B, Isolated from Soil”

**DOI:** 10.1128/MRA.01080-21

**Published:** 2021-11-24

**Authors:** Jackson Harris, Marie Gross, Jeremy Kemball, Sanaz Farajollahi, Patrick Dennis, John Sitko, J. Jordan Steel, Erin Almand, Nancy Kelley-Loughnane, Vanessa A. Varaljay

**Affiliations:** a Department of Biology, United States Air Force Academy, Colorado Springs, Colorado, USA; b UES, Inc., Dayton, Ohio, USA; c University of Dayton, Dayton, Ohio, USA; d Soft Matter Materials Branch, Materials and Manufacturing Directorate, Air Force Research Laboratory, Wright-Patterson Air Force Base, Ohio, USA

## ERRATUM

Volume 10, no. 44, e00635-21, 2021, https://doi.org/10.1128/MRA.00635-21. Page 1, lines 7–10: “The sequenced genome of *D. acidovorans* strain D4B provides the first genome for an isolate of this genus, which will further our understanding of how this organism could be leveraged for bioremediation.” should read “The sequenced genome of *D. acidovorans* strain D4B will further our understanding of how this organism can be leveraged for bioremediation.”

